# MicroRNA-181 regulates the development of Ossification of Posterior longitudinal ligament via Epigenetic Modulation by targeting PBX1

**DOI:** 10.7150/thno.44309

**Published:** 2020-06-12

**Authors:** Ning Liu, Zicheng Zhang, Li Li, Xiaolong Shen, Baifeng Sun, Ruizhe Wang, Huajian Zhong, Qianghui Shi, Leixin Wei, Yizhi Zhang, Yue Wang, Chen Xu, Yang Liu, Wen Yuan

**Affiliations:** 1 Department of Orthopedics, Changzheng Hospital Affiliated to Second Military Medical University, 415th Feng Yang Road, Shanghai, 200003, PR China.; 2 Undergraduate Brigade, Changhai Hospital Affiliated to Second Military Medical University, 168th Chang Hai Road, Shanghai, 200433, China.; 3 Research Center of Developmental Biology, Second Military Medical University, 800th Xiang Yin Road, Shanghai, 200433, PR China.

**Keywords:** OPLL, miR-181, PBX1, ossification, histone modification

## Abstract

**Objectives:** Ossification of the posterior longitudinal ligament (OPLL) presents as the development of heterotopic ossification in the posterior longitudinal ligament of the spine. The etiology of OPLL is genetically linked, as shown by its high prevalence in Asian populations. However, the molecular mechanism of the disease remains obscure. In this study, we explored the function and mechanism of OPLL-specific microRNAs.

**Methods:** The expression levels of the ossification-related OPLL-specific miR-181 family were measured in normal or OPLL ligament tissues. The effect of miR-181a on the ossification of normal or pathogenic ligament cells was tested using real-time polymerase chain reaction (PCR), Western blot, alizarin red staining and alkaline phosphatase (ALP) staining. The candidate targets of miR-181 were screened using a dual luciferase reporter assay and functional analysis. The link between miR-181a and its target PBX1 was investigated using chromatin immunoprecipitation, followed by real-time PCR detection. Histological and immunohistochemical analysis as well as micro-CT scanning were used to evaluate the effects of miR-181 and its antagonist using both tip-toe-walking OPLL mice and *in vivo* bone formation assays.

**Results:** Using bioinformatic analysis, we found that miR-181a-5p is predicted to play important roles in the development of OPLL. Overexpression of miR-181a-5p significantly increased the expression of ossification-related genes, staining level of alizarin red and ALP activity, while the inhibition of miR-181a-5p by treatment with an antagomir had the opposite effects. Functional analysis identified PBX1 as a direct target of miR-181a-5p, and we determined that PBX1 was responsible for miR-181a-5p's osteogenic phenotype. By chromatin immunoprecipitation assay, we found that miR-181a-5p controls ligament cell ossification by regulating PBX1-mediated modulation of histone methylation and acetylation levels in the promoter region of osteogenesis-related genes. Additionally, using an *in vivo* model, we confirmed that miR-181a-5p can substantially increase the bone formation ability of posterior ligament cells and cause increased osteophyte formation in the cervical spine of tip-toe-walking mice.

**Conclusions:** Our data unveiled the mechanism by which the miR-181a-5p/PBX1 axis functions in the development of OPLL, and it revealed the therapeutic effects of the miR-181a-5p antagomir in preventing OPLL development both *in vivo* and *in vitro*. Our work is the first to demonstrate that microRNA perturbation could modulate the development of OPLL through epigenetic regulation.

## Introduction

Ossification of the posterior longitudinal ligament (OPLL) is a relatively common spinal degenerative disease that can lead to severe neurological symptoms as a result of spinal cord compression, which is caused by the gradual enlargement of heterotopic ossification (HO) of the posterior longitudinal ligament of the spine 1. OPLL most commonly occurs in the cervical spine, and an epidemiological study showed that the prevalence of OPLL mainly clustered in Asian populations, with an incidence of 1.9-4.3% in Japan and other Eastern countries 2. In recent years, a comparable incidence has also been reported in other countries; further, the average age of onset is becoming younger, and it occurs predominantly in men 3. The clinical presentation of OPLL is primarily myelopathy with or without radiculopathy, and OPLL patients often have serious neurological pathology, resulting in paralysis of extremities and disturbances of motility that decrease the quality of life. Patients with symptomatic OPLL usually develop large osteophytes that require surgical treatment by means of direct or indirect decompression via an anterior or posterior approach that has now become the standard for treating the disease 4. However, even after surgery, ossification can gradually progress over time, which may again cause symptoms. Thus, finding a way to suppress osteophyte progression could be a complementary or even preventive treatment for OPLL patients.

Current nonsurgical management options for OPLL consist of physical therapy, observation, and administration of oral analgesics 1. However, these approaches are used only for symptomatic relief, and no specific treatment has been developed for curing or preventing the ossification of the spinal ligament until now. This phenomenon is largely due to the poor understanding of OPLL pathophysiology. Although multiple studies have indicated an underlying genetic etiology, the incomplete understanding of the mechanisms by which osteogenesis takes place in the posterior longitudinal ligament of OPLL patients has greatly impeded gaining more insight into the disease 3, 5, 6. Previous studies on OPLL have focused on genetic variants and have suggested that osteogenic factors such as *COL1A2, NNPs, TGF-β1, BMP-2/4, COL6A1,* and *RUNX2* are associated with the susceptibility to and severity of OPLL. However, the detailed mechanism behind OPLL is unknown. These factors have been identified as contributing to normal skeletal development or related diseases 6-8. Thus, we speculate that these factors are important “effectors” rather than “activators” in OPLL development. In our previous studies, using high throughput technology, we identified OPLL-specific microRNAs and deciphered their regulatory network 9, but their specific roles in OPLL requires further validation.

MicroRNAs (miRNAs) are small (20-25 nucleotides long) noncoding RNAs that regulate gene expression in numerous biological or pathological processes by binding with target mRNAs to affect their translation or stability, and they are thought to regulate cell reprogramming and differentiation 10, 11. Approximately 30%-80% of mRNAs in humans are regulated by miRNAs at the posttranscriptional level. Our previous studies have identified the important role and mechanism of miR-10a-3p in OPLL 12. However, as a result of the complex and dynamic interactions within this miRNA regulation network, the *in vivo* functional role of miRNAs in OPLL patients has yet to be validated.

In this study, taking advantage of bioinformatic analysis of our previous high-throughput sequencing data, we identified another important ossification-related microRNA, microRNA-181a, which may play an active role in OPLL development. Through functional analysis, we demonstrated that microRNA-181a directly targets PBX1 to regulate OPLL development through epigenetic modification of RUNX2 and other ossification-related genes. Moreover, for the first time, we validated the effect of miR-181a-5p on OPLL development and bone formation using an *in vivo* tip-toe-walking OPLL mouse model, and we showed that a miR-181a-5p antagonist may be functional in preventing OPLL development.

## Methods

### Primary cell culture and sample collection

All experimental protocols were approved by the Ethics Committee of Second Military Medical University. Ligament samples were obtained from participants who provided written informed consent, and all related methods were carried out in accordance with the approved guidelines. The diagnosis of OPLL or PLL (spinal trauma patients who underwent cervical corpectomy) was confirmed in our institution by computerized tomography (CT) and magnetic resonance imaging preoperatively. OPLL or PLL specimens were obtained from primary intraoperative tissues of patients undergoing surgery and were immediately used in primary cell culture as previously described 12. In brief, small pieces of ligament tissue were plated on culture dishes with Dulbecco's Modified Eagle Medium (DMEM, Life Technology Gibco, USA) supplemented with 10% fetal bovine serum (FBS, Gibco, USA), 1% L-glutamine (Gibco, USA), and 1% penicillin/streptomycin (Gibco, USA); the pieces were then incubated at 37°C in a humidified atmosphere containing 95% air and 5% CO_2_. The fibroblast-like spindle-shaped cells without cuboidal or flattened morphology that migrated from the tissue pieces were considered as ligament cells and were harvested for further expansion and analysis. Altogether, 22 OPLL patient tissue samples (13 men and 9 women, aged 45-72 years, mean age 57.7 years) and 19 PLL patient samples (11 men and 8 women, aged 44-69 years, mean age 55.2 years) were collected during anterior cervical corpectomy, and the detailed information of the patients is listed in Supplementary Table 1 (Table S1).

### MiRNA/mRNA interactive network construction

The miRNAs that were differentially expressed between PLL and OPLL were identified in our previous study using parameters of P≤0.01 and fold change ≥2 or ≤0.5 9. The processed data were used to generate an ossification-related interactive network. For miRNA prediction, TargetScan (www.targetscan.org) was used to predict the binding of differentially expressed miRNAs to their putative mRNA targets. The predicted target genes were compared with the transcriptome profiling data of GSE69787 to establish a miRNA/mRNA negative correlation, and only genes that were categorized according to the GO term Ossification (GO:0001503) were kept for the network construction. The related data are listed in Supplementary Data 1.

### Lentiviral construction and transfection

For PBX1 overexpression, *in vivo* PBX1 hairpin knockdown, *in vivo* miR-181a-5p overexpression and *in vivo* miR-181a-5p inhibition, we took advantage of lentivirus and constructed lentiviral vectors for overexpression and short hairpin RNA (shRNA) mediated knockdown. The PBX1 CDS sequence was synthesized and cloned into pLenti-CMV-MCS-PGK-mCherry-T2A-Puro vector, PBX1 hairpin knockdown sequence was synthesized and cloned into pLKD-CMV-Puro-U6-shRNA vector, mature miR-181a sequence was synthesized and cloned into pLenti-U6-mir-181a-EF1a-Puro vector and miR-181a-5p inhibition sequence (8 × miR-181a-5p antisense sequence as miR-181a-5p sponge to inhibit miR-181a-5p expression) was synthesized and cloned into pLOV-EF1a-PuroR-CMV-EGFP-3FLAG-Sponge vector (all performed by Obio Technology Corp.,Lid, Shanghai, China). All plasmids were either entirely or partially sequenced to ensure fidelity before lentivirus packaging. For lentivirus transfection, the lentiviruses were added the second day of cell passage at multiplicity of infection (MOI) of 10 with 6 µg/ml of polybrene (Obio Technology Corp.,Lid, Shanghai, China), and the medium containing lentivirus were changed after 48h.

### Oligonucleotide syntheses and transfection

For miRNA overexpression and inhibition, miRNA Agomirs (antisense strand chemically modified miRNA mimics with 3' terminal cholesterol modification, two thiols modification in the 5' terminal, four thiols modification in the 3' terminal, and the whole strand is modified by OMe) and Antagomirs (3' terminal modified by cholesterol, two thiols modification in the 5' terminal, four thiols modification in the 3' terminal, and the whole strand is modified by 2'-OMe) were synthesized by GenePharma Corp (Shanghai, China). Agomirs and Antagomirs are all designed based on the mature microRNA sequence. A scramble miRNA mimic control was used as negative control. For PBX1 and ACAN silencing, two siRNAs for each gene were designed and synthesized by GenePharma Corp with 2'-Ome modification, and were combined to achieve better silencing effect. A scramble siRNA control that targets none of the PBX1 and ACAN mRNA was used as negative control. The indicated Agomirs, Antagomirs, siRNAs or scramble controls were commonly transfected at a final concentration of 20 pmol/ml if not mentioned. The transfection was taken out using Lipofectamine RNAiMAX (Thermo Fisher, USA) according to the manufacturer's protocol. The transfections were performed 24h after cell passage, and the culture medium containing the transfection reagent were changed after 12h of transfection. Cells were collected 48h after the transfection for further analysis. The related sequences used were listed in Supplementary Data 1.

### Dual-Luciferase reporter assay

For dual luciferase report assay, the Reporter constructions were performed by synthesizing wild type or mutated 3'UTR of PBX1 and ACAN and subcloned into pMIR-REPORT vector (Promega, WA, USA), which all these procedures were done by GeneChem Corp (Shanghai, China). Before the experiments, the HEK293T cells were seeded in 96-well plates for 24 hours, and a mixture of the pMIR-REPORT vector (wild type or site mutated plasmid) and miRNAs mimics or scramble control were co-transfected into cells, and a PRL-TK vector (carrying Renilla luciferase) was also co-transfected and served as internal control (Promega, Madison, USA). The transfection uses Lipofectamine 2000 (Invitrogen) with 50 ng of pMIR-REPORT vector (carrying firefly luciferase), 25ng of PRL-TK vector (carrying Renilla luciferase) and 10nmol of miRNA mimics or scramble controls. After transfection for 48 hours, the Dual-Luciferase Reporter Assay System (Promega, Madison, USA) was used to detect the luciferase activity. And Light intensity was normalized by Firefly luciferase.

### RNA extraction and real-time qPCR

Cell samples were washed twice with PBS and then lysed with 750 μL of Trizol (Invitrogen, Carlsbad, USA) per sample for total RNA extraction. For tissue samples, chunks of approximately 3 × 3 × 3-mm tissue material were homogenized in 750 μL of Trizol per sample. Total RNA was then extracted according to the manufacturer's instruction and further reverse transcribed using ReverTra Ace® qPCR RT Kit (Toyobo, Osaka, Japan). Real-time PCR was performed and analyzed as previously described 11. Single strand cDNA was analyzed with SYBR Green master mix (Roche, USA) according to the manufacturer's instructions, and the primer sequences used in this study are listed in Supplementary Data 2.

### Alizarin red, Alkaline phosphatase staining and quantification

To determine the osteogenic properties of ligament cells, Alizarin red S staining (Sciencell, San Diego, USA) and ALP activity assay (Sidansai, Shanghai, China) were performed 3 weeks after osteogenic induction as previous described 12. Briefly, cells were treated with osteogenic induction medium consisting of DMEM with 10% FBS, 25 mg/ml ascorbate-2 phosphate, 10^-8^M dexamethasone, and 5mM β-glycerophosphate (All from Gibco, USA) for the 2 weeks. After induction, cells were cells were fixed with 4% paraformaldehyde and washed with phosphate buffered solution (PBS), then stained according to the manufacturer's instruction.

### Immunohistochemistry analysis

The specimens were decalcified in 10% ethylene diamine tetraacetic acid (EDTA, pH 7.4) for 1 month, followed by dehydration and embedding in paraffin. Sections (5 μm) were cut and stained with hematoxylin and eosin (H&E). For immunohistochemistry analysis, sections were blocked with 3% BSA for 30 min and then incubated with primary antibody against PBX1 (ab97994, Abcam, USA) and OCN (23418-1-AP, ProteinTech, Wuhan, China) were used at 1:100 dilutions. Antigen retrieval was performed in 95°C citrate buffer (pH 6) for 10 min, then sections incubated with primary antibody at 4°C overnight. After processed by the ABC detection kit (Vector Laboratories, Burlingame, CA), sections were visualized under an Olympus BX51 light microscope equipped with Olympus DP70 camera (Olympus Co., Tokyo, Japan) and quantified using the ImageJ software (US National Institutes of Health).

### Western Blots

Proteins were extracted using a commercial kit (No.C510003, Sangon Biotech, China) according to the manufacturer's instructions. Primary antibodies rabbit anti-RUNX2 (ab23981, Abcam), rabbit anti-OSX (ab94744, Abcam), rabbit anti-ACAN (ab36861, Abcam), rabbit anti-OCN (23418-1-AP, ProteinTech, Wuhan, China), rabbit anti-ALP (ab83259, Abcam) and rabbit anti-PBX1 (ab97994, Abcam) were used (all at 1:1,000 dilution). The protein samples were separated on 10% SDS-PAGE gels and subsequently transferred to nitrocellulose filter membranes (Pall Corp.,Washington, NY) using the wet transfer blotting system (BioRad, Hercules, CA). After incubation, secondary antibody goat anti-rabbit-HRP (Pierce, USA) was used at 1:2,000 dilution. The proteins were then detected by chemiluminescence detection system (Millipore, USA). Anti-GAPDH was used as an endogenous control.

### Chromatin immunoprecipitation analysis

Chromatin immunoprecipitation was performed using EZ-Magna ChIP A/G Kit (Millipore, Billerica, MA, USA) following the manufacturer's instructions. Briefly, PLL/OPLL cells transfected with miR-181a-5p agomirs or antagomirs were fixed using formaldehyde with final concentration of 1% to perform cross-linking after incubation for 20 minutes at room temperature, glycine was added to terminate the process at final concentration of 0.2M. Cells were harvested and lysed by lysis buffer supplemented with protease inhibitors, and the lysates were collected for sonification (4 cycles of 25s on, 59 s off, pulse at a power output of 20%) at 4°C. For immunoprecipitation, the samples were incubated with rabbit anti-PBX1 (ab97994, Abcam), anti-RUNX2 (ab23981, Abcam), anti-HOXA10 (ab175026, Abcam), anti-H3K9ac (ab4441, Abcam), anti-H2K9me2 (ab32521, Abcam), anti-RNAPol II (ab5095, Abcam) or a negative rabbit IgG antibody overnight at 4°C, which was followed by incubation with protein A/G agarose resin. The purified immunoprecipitated DNA was further quantified by real-time PCR analysis. The information for all primers is listed in Supplementary Data 2.

### *In vivo* heterotopic bone formation assay and OPLL model analysis

For *in vivo* heterotopic bone formation assay, we transfected PLL cells with miR-181a-5p overexpressing lentivirus or inhibitor overexpressing lentivirus or control lentivirus for 4 days and resuspended the ligament cells to co-culture with Bio-Oss Collagen (Geistlich, GEWO GmbH, Baden, Germany) scaffold in osteogenic medium for 2 days. Then the seeded scaffolds were implanted subcutaneously on the back of 4-week-old BALB/c homozygous nude (nu/nu) mice (6 mice per group) as described previously 12. Six weeks later, the scaffolds were harvested and fixed in 4% paraformaldehyde for further analysis.

To assess the OPLL formation *in vivo*, we introduced tip-toe walking mice (TWY-ttw mice) for the experiment (Central Institute for Experimental Animals, CIEA, Kawasaki, Japan). 6-week old ttw mice were used in this study, and miR-181a-5p agomirs or antagomirs were injected into the tail vein at a concentration of 20nmol per mouse. The injection repeated at 2-week interval until 2 months after. All mice were sacrificed after 18 weeks, unless mice die during the experiment. The spine of all mice was harvested and submitted to micro-CT scanning and Immunohistochemistry analysis. During the experiment, mice were daily observed for limb spasms during movement, weaknesses in limbs and abnormal gait which would indicate several compressions in the spine canal (Supplementary Video 1). All animal experiments were approved by the Second Military Medical University Animal Care and Use Committee.

### Micro-CT analysis

Bio-scaffold or spine of *ttw* mouse were harvested after treatment and fixed in 4% neutral-buffered formalin, Micro-CT scanning and analysis was performed by Shanghai Model Organisms Center Inc (Siemens, Munich, Germany). Micro-CT image was processed by Inveon Research Workplace (Siemens Healthcare GmbH, Erlangen, Germany) to acquire 3D reconstruction, volume quantification. The region of interest was selected within the bio-scaffold to calculate bone volume (CT value above 2000Hu)/total volume (CT value above 700Hu), bone mineral density (BMD, mg/ml). Spinal CT images with significant OPLL were used to calculate occupation percentage of the spinal canal.

### Statistical analysis

Data are reported as mean values ± SD (standard derivation). Data analysis was performed using SPSS for Windows version 16.0. Student's t test and one-way ANOVA were performed where appropriate and a P value <0.05 was considered as statistically significant.

## Results

### MicroRNA-181a is a potential key regulator of ossification in OPLL

**Figure 1:**
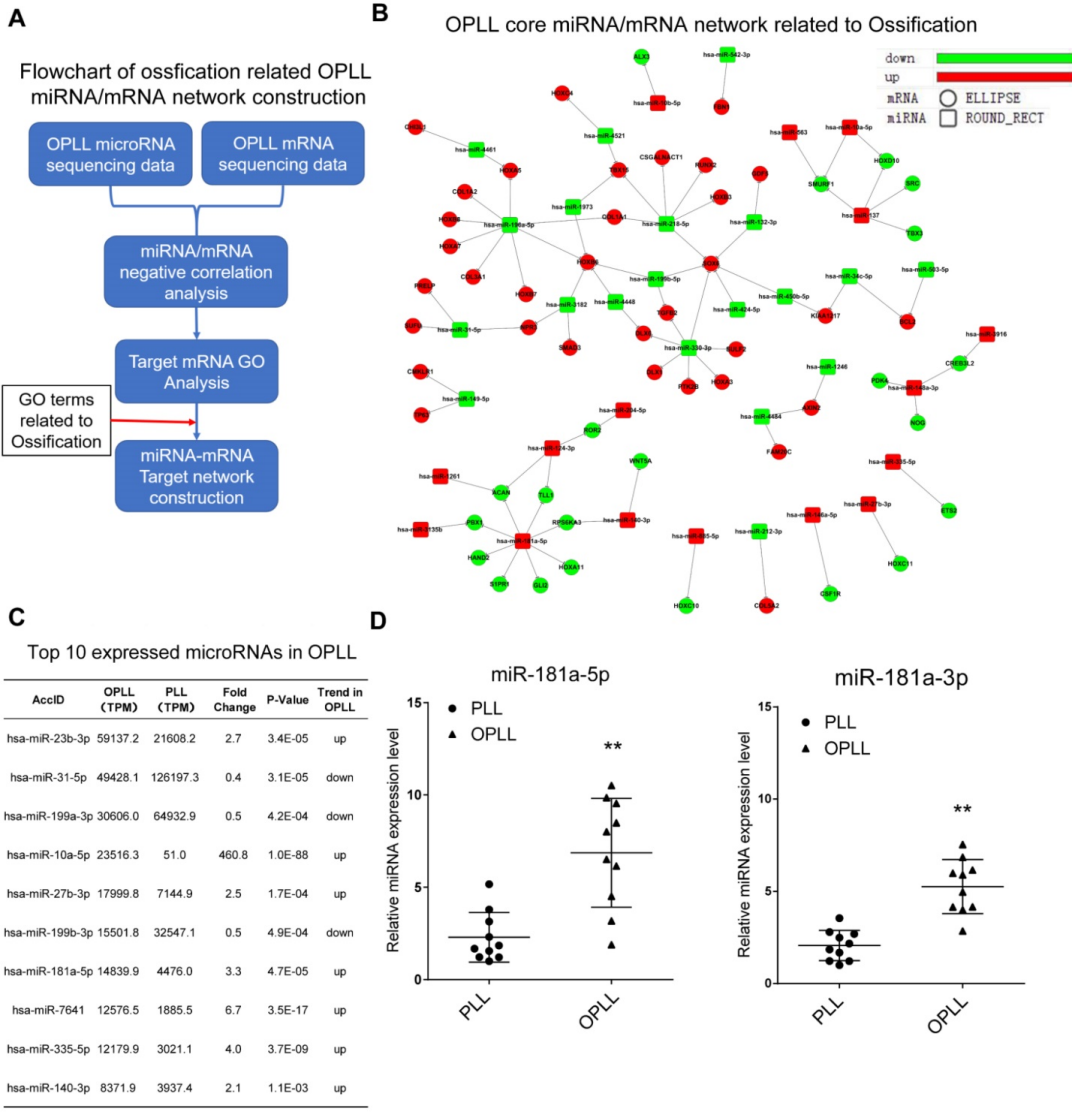
Ossification related miR-181a is a potential factor in association with OPLL. (A) The flowchart of ossification related OPLL miRNA/mRNA negative regulatory network construction. (B) The miRNA/mRNA interactive network related to ossification in OPLL. Ellipse shape dot represents differentially expressed mRNAs, and rectangle shape dot represents differentially expressed miRNAs. Upregulated genes in OPLL were labeled red, and downregulated labeled green. (C) A List of top 10 miRNA expressed in OPLL. miR-181a-5p is the seventh highest ranked miRNA in OPLL based on GEO dataset GSE69787. (D) Expression validation of miR-181a in PLL (n=10) and OPLL (n=10) tissues determined by real-time PCR. The horizontal lines represent mean and quartile values. **P < 0.01.

In a previous study, our group first revealed the microRNA regulatory network of OPLL 9. However, further validation is needed to unveil the specific mechanism of each potential gene in the network. In our previous study, we found that ligament cells, especially those from OPLL patients, can be effectively induced into an osteoblast phenotype 12. Therefore, we first tried to find the key component that is responsible for the ossification transformation of the posterior longitudinal ligament using bioinformatic analysis of existing high-throughput RNA sequencing data (GEO dataset GSE69787, Figure 1A). Using the previous data, we constructed an ossification-related miRNA/mRNA negative regulatory network by including only genes that were categorized in the Gene Ontology term 'Ossification' (GO:0001503, Figure 1B). We found that miR-218-5p, miR-196a-5p, miR-330-3p and miR-181a-5p had more clustered ossification-related targets than other microRNAs, which implies a potential role for these microRNAs in OPLL development. Because miRNAs must be maintained at high intracellular levels to exert a significant effect 13, we further narrowed down the microRNAs to identify a priority candidate by examining their expression levels in OPLL. We found that of the differentially expressed miRNAs in the network we constructed, miR-181a-5p was highly expressed in the OPLL ligament cells and was upregulated significantly when compared to normal posterior longitudinal ligament cells (Figure 1C). Furthermore, using OPLL and normal tissue samples, we confirmed that the expression of miR-181a-5p and its counterpart miR-181a-3p was upregulated in OPLL tissue (Figure 1D), so we chose miR-181a as a candidate microRNA for further analysis.

### miRNA-181a-5p modulates the osteogenic property of posterior longitudinal ligament cells

**Figure 2:**
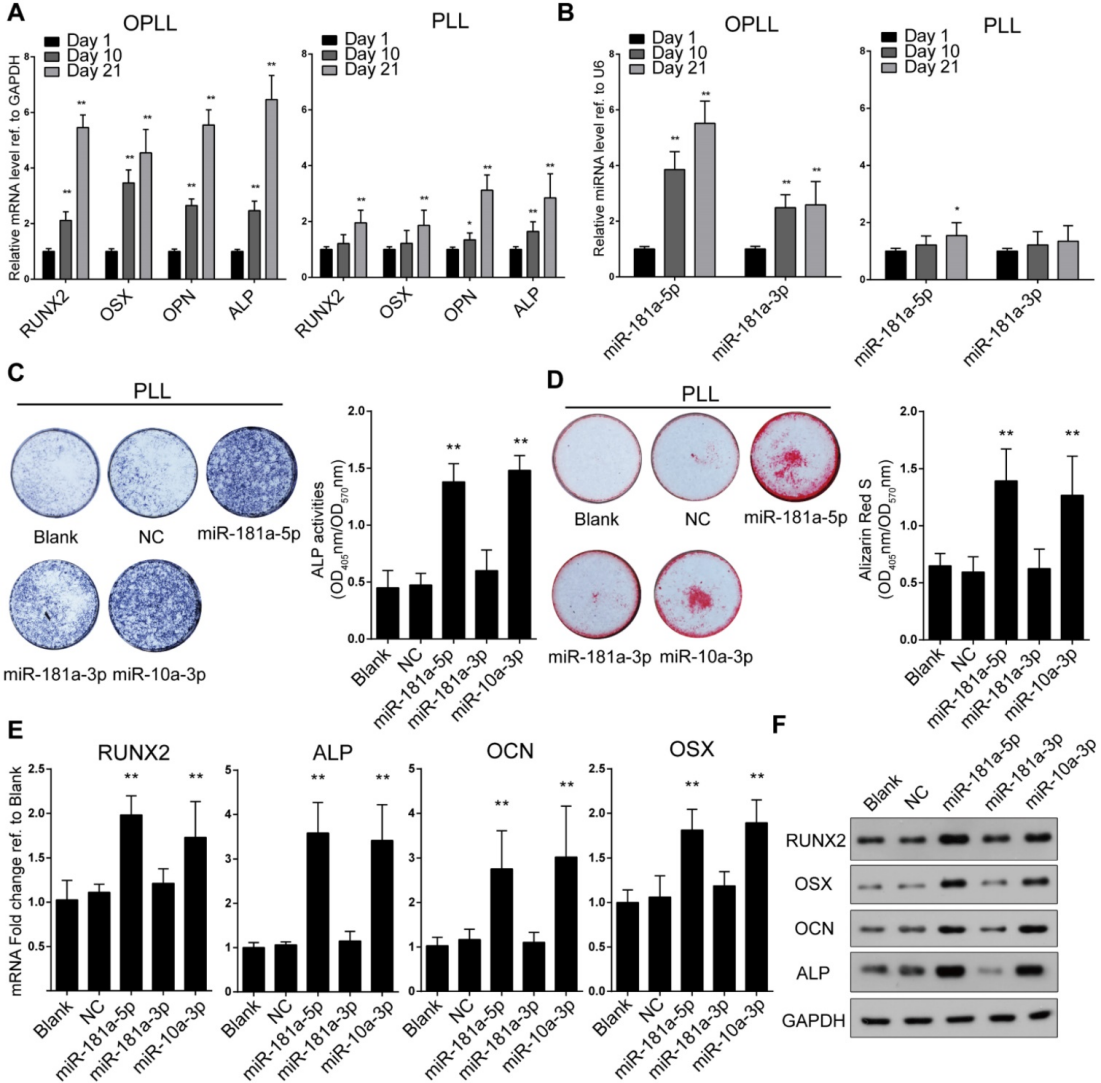
miR-181a-5p overexpression increases osteogenic property of PLL cells. (A) mRNA expression levels of ossification-related genes at different time point of osteogenic induced OPLL or PLL cells detected by real-time PCR. (B) miRNA expression levels of miR-181a-5p and -3p are detected by real-time PCR at different time point of osteogenic induced OPLL or PLL cells. The osteogenic properties of PLL cells are analyzed using alkaline phosphatase staining (C) or alizarin red staining (D) after osteo-induction for 21 days. The colorimetric quantification is shown in the right panels, respectively. NC group represents transfecting scramble control miRNA mimics. The quantification of expression of ossification related genes were detected using either real-time PCR (E) or Western Blot (F) under the same condition. All PCR experiments were repeated three times individually, and GAPDH level or U6 level were detected and served as internal reference. All data were presented as the mean ± SD. *P < 0.05, **P < 0.01.

To further analyze the effect and mechanism of miR-181a in OPLL, we made use of OPLL and normal PLL primary ligament cells from patients. The outgrowth cells that exhibited fibroblastic-like spindle-shaped cells without cuboidal or flattened morphology were considered ligament cells, although they differed in size between OPLL and PLL primary ligament cells, which we described previously 9. Initially, we found that the RNA levels of miR-181a-5p and miR-181a-3p were highly expressed in OPLL cells (Figure S1A), and the level of miR-181a-5p was significantly higher than that of miR-181a-3p in OPLL cells (Figure S1B). The osteogenic properties of OPLL and PLL ligament cells were confirmed by examining the mRNA levels of ossification-related genes (RUNX2, OSX, OCN, and ALP) at different time points during osteogenic induction (Figure 2A). Consistent with previous findings, OPLL cells showed greater osteogenic properties than PLL cells. Additionally, we found that the levels of miR-181a-5p and miR-181a-3p were significantly upregulated in OPLL cells after osteogenic induction but not in PLL cells (Figure 2B), which indicates a vital role in OPLL ligament ossification. To investigate the function of miR-181a, we overexpressed miR-181a-5p and miR-181a-3p using microRNA mimics (Figure S1C), and we found that miR-181a-5p could significantly promote the ALP activity and calcium deposition of PLL cells after osteoinduction, while miR-181a-3p overexpression had a smaller effect (Figure 2C-D). We also confirmed the osteogenic promotion effect of miR-181a-5p by analyzing the expression level of the ossification-related genes RUNX2, OSX, OCN and ALP using both real-time PCR and Western blot experiments; the results showed that miR-181a-5p overexpression was able to upregulate the expression of ossification-related genes at both the RNA and protein levels (Figure 2E-F). The effect of miR-181a-5p was comparable to that of miR-10a-3p, a previously reported microRNA that correlates with OPLL development 12.

**Figure 3:**
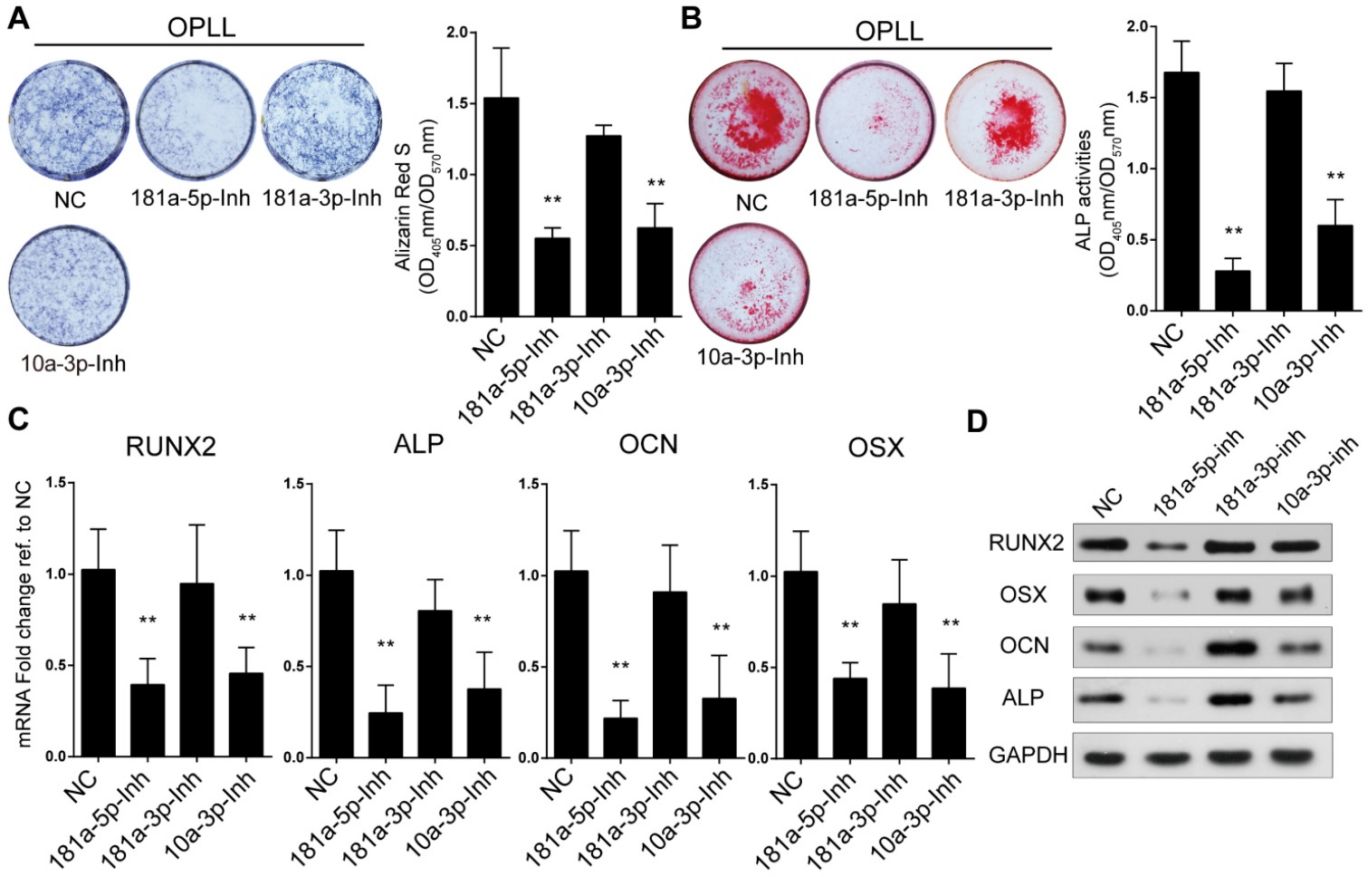
Inhibition of miR-181a-5p decreases osteogenic property of OPLL cells. Alkaline phosphatase staining (A) or alizarin red staining (B) were used to analysis osteogenic properties of miR-181a-5p inhibition (181a-5p-Inh), miR-181a-3p inhibition (181a-3p-Inh) and miR-10a-3p inhibition (10a-3p-Inh) in OPLL cells after osteo-induction for 21 days. The colorimetric quantification is shown in the right panels, respectively. NC group represents transfecting scramble control miRNA mimics. Ossification-related genes are assessed by real-time PCR (C) and Western Blot (D) after osteo-induction for 21 days under the same conditions respectively. All PCR experiments were repeated three times individually, and GAPDH level were detected and served as internal reference. All data were presented as the mean ± SD. *P < 0.05, **P < 0.01.

The importance of miR-181a was also analyzed using modified miRNA antisense inhibitors (antagomirs). Knockdown of expression of miR-181a-5p, miR-181a-3p and miR-10a-3p was performed by transfecting the antagomirs into OPLL cells (Figure S1D). We analyzed antagomir-treated OPLL cells using the same approaches and found that inhibition of both miR-181a-5p and miR-10a-3p, but not miR-181a-3p, showed a significant reduction in alizarin red staining and ALP activity in OPLL cells after osteogenic induction (Figure 3A-B). Similar results were obtained by analyzing the expression levels of RUNX2, OSX, OCN and ALP. We found that inhibition of both miR-181a-5p and miR-10a-3p downregulated the expression of ossification-related genes (Figure 3C-D). Taken together, we found that miR-181a-5p, but not miR-181a-3p, could strongly regulate the ossification of both PLL and OPLL cells *in vitro*.

### PBX1 and ACAN are targets of miR-181a-5p in OPLL

**Figure 4:**
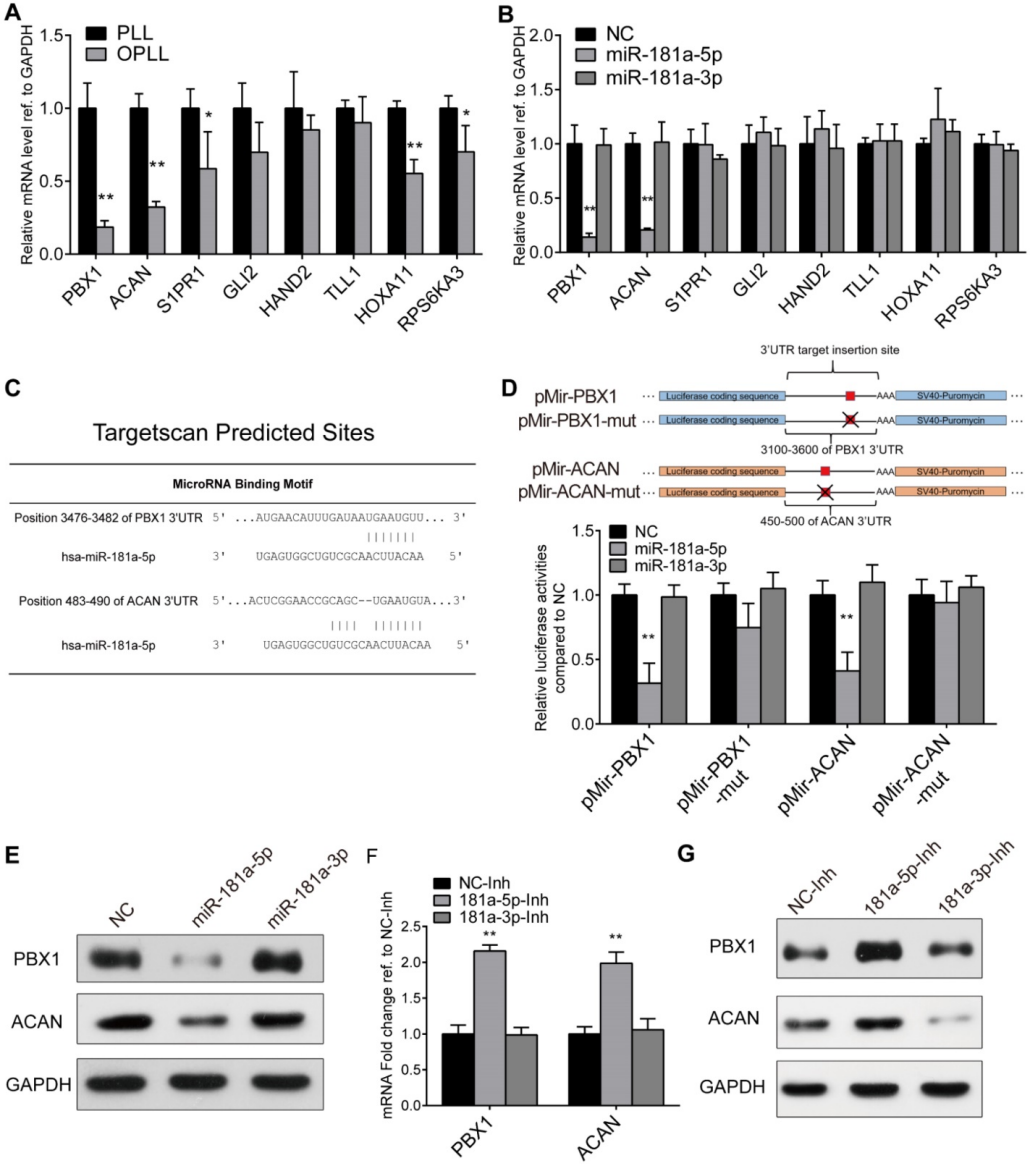
PBX1 and ACAN are targeted by miR-181a-5p. (A) Real-time PCR analysis detecting the mRNA expression levels of miR-181a-5p predicted targets in PLL and OPLL cells. (B) Real-time PCR analysis detecting the mRNA expression levels of miR-181a-5p predict targets after miR-181a-5p or -3p overexpression in PLL cells. (C) Targetscan prediction of the miR-181a-5p binding motif to PBX1 and ACAN 3`UTR. (D) Dual luciferase reporter assay detecting the activities of firefly luciferase generated by respective 3'UTR bearing plasmids after miR-181a-5p or -3p overexpression in HEK293T cells (n=6). (E) Western Blot analysis showing the protein levels of PBX1 or ACAN after miR-181a-5p or -3p overexpression in PLL cells. (F) mRNA levels of PBX1 and ACAN after miR-181a-5p or -3p inhibition in OPLL cells using real-time PCR analysis. (G) Western Blot analysis showing the protein levels of PBX1 or ACAN after inhibition of miR-181a-5p or -3p in OPLL cells. All PCR experiments were repeated three times individually, and GAPDH level were detected and served as internal reference. All data were presented as the mean ± SD. *P < 0.05, **P < 0.01.

Next, we attempted to identify a direct target of miR-181a-5p to clarify the underlying mechanism during OPLL development. Taking advantage of our previous sequencing data and miRNA target prediction network (Figure 1B), we first tried to narrow down possible candidates by determining the expression change of predicted targets between OPLL and PLL ligament cells using real-time PCR (Figure 4A). Furthermore, we directly analyzed the effect of miR-181a-5p on these targets *in vitro* by transfecting cells with microRNA mimics (Figure 4B). The results showed that among the 8 predicted target genes, the expression of PBX1 and ACAN was significantly decreased in OPLL cells after miR-181a-5p transfection (Figure 4A-B), thus indicating that PBX1 and ACAN are potential targets of miR-181a-5p.

To confirm this, we performed a luciferase reporter assay to test whether miR-181a-5p could directly affect the expression of PBX1 or ACAN through a posttranscriptional repression mechanism. Luciferase reporter plasmids encoding the predicted 3′UTR region of PBX1 or ACAN mRNA in wild-type or site-mutated (mut) form were used in the test (Figure 4C-D). miRNAs were cotransfected along with the plasmids into HEK-293T cells. The luciferase activities of both the pMir-PBX1 and pMir-ACAN groups showed significant downregulation after miR-181a-5p overexpression, while miR-181a-3p did not show a significant effect (Figure 4D). In the site mutated group, no significant reduction of luciferase activities was observed after miR-181a-5p overexpression (Figure 4D). The effect of miR-181a-5p on PBX1 and ACAN was also validated at the protein level (Figure 4E). We again used real-time PCR and Western blot experiments to demonstrate that the inhibition of miR-181a-5p in OPLL cells could significantly upregulate the expression of PBX1 and ACAN (Figure 4F-G). Collectively, these results support the premise that miR-181a-5p, but not miR-181a-3p, directly targets and negatively regulates PBX1 and ACAN in ligament cells.

### miR-181a-5p targeting of PBX1 is necessary for its promotion of osteogenesis in OPLL cells

**Figure 5:**
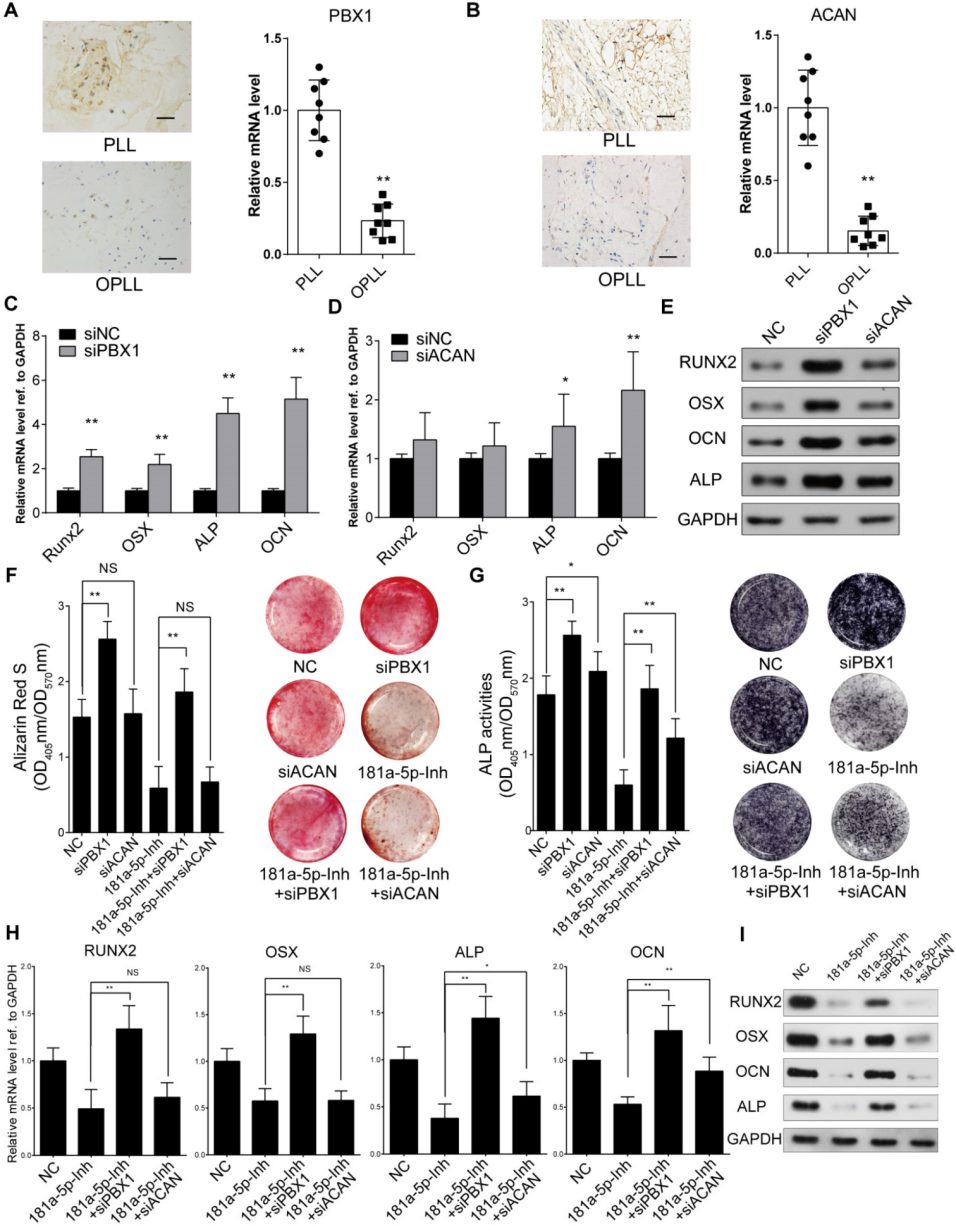
PBX1 is essential for miR-181a-5p to regulate osteogenesis in ligament cells in OPLL. *In situ* hybridization histochemistry is used to analysis the expression level of PBX1 (A) or ACAN (B) in PLL and OPLL patients' tissue (n=6). The bar represents 100μm. Real-time PCR analysis showing the mRNA expression level of osteogenic genes after knockdown of PBX1 (C) or ACAN (D) using small interference RNAs in OPLL cells. The siNC represents transfecting scramble control siRNAs which serve as control group. (E) The protein levels of osteogenic genes after knockdown of PBX1 or ACAN in OPLL cells were detected using Western Blot analysis. Alizarin red staining (F) or alkaline phosphatase staining (G) analysis to compare the osteogenic properties of respective treatment in OPLL cells. The colorimetric quantification is shown in the right panels, respectively. (H) Real-time PCR analysis was used to analysis mRNA expression levels of osteogenic genes in various groups in OPLL cells. (I) The protein levels of osteogenic genes after respective treatment in OPLL cells were detected using Western Blot analysis. All PCR experiments were repeated three times individually, and GAPDH level were detected and served as internal reference. All data were presented as the mean ± SD. *P < 0.05, **P < 0.01.

To specifically examine the role of PBX1 and ACAN in miR-181a-5p-mediated OPLL development, we first performed *in situ* hybridization histochemistry using posterior longitudinal ligament tissue from PLL and OPLL patients (n=8). The colorimetric quantification showed that the relative expression levels of PBX1 and ACAN were significantly lower in OPLL tissues than they were in PLL tissues (Figure 5A-B). We tried to test the function of PBX1 and ACAN in osteogenic induction of ligament cells by knocking them down using small interfering RNA oligos (siRNAs, Figure S2A-B). As shown by Western blot and real-time PCR analysis, transfection of siRNAs targeting PBX1 in OPLL cells dramatically upregulated the expression of RUNX2, OSX, ALP and OCN, while knockdown of ACAN only significantly downregulated the expression levels of ALP and OCN (Figure 5C-E). In PLL cells, knockdown of ACAN showed no significant changes in the expression of ossification-related genes (Figure S2C-D). Furthermore, we tested the role of PBX1 and ACAN in miR-181a-5p-mediated OPLL cell ossification. We co-transfected OPLL cells with a miR-181a-5p inhibitor and a PBX1 siRNA or an ACAN siRNA and then subjected them to osteo-induction for 21 days. Alizarin red staining and alkaline phosphatase assay results showed that knockdown of PBX1 expression significantly increased the ossification phenotype in OPLL cells, while ACAN knockdown only showed mild upregulation of ALP activity in OPLL cells (Figure 5F-G). Additionally, only PBX1 knockdown significantly reversed the reduced mineral deposition and decreased the ALP activity of OPLL cells upon miR-181a-5p inhibition (Figure 5F-G). Real-time PCR and Western blot analysis was used to analyze the expression level of ossification-related genes, and the data further confirmed the role of PBX1 in miR-181a-5p-mediated OPLL cell ossification (Figure 5H-I).

To confirm these findings, we performed lentiviral-mediated PBX1 overexpression in OPLL cells (Figure S3A). We found that after PBX1 overexpression, the relative expression level of osteogenic genes was significantly downregulated (Figure S3B), which is consistent with previous findings 14. Further, alizarin red staining and alkaline phosphatase assay results showed that PBX1 overexpression could reverse the osteogenic promotion effect of miR-181a-5p overexpression in osteogenic-induced OPLL cells (Figure S3C-D). Real-time PCR analysis was used to analyze the expression level of ossification-related genes, and the data also confirmed the role of PBX1 in miR-181a-5p-mediated OPLL cell ossification (Figure S3E). PBX1 did not affect the expression levels of ACAN, miR-10a-3p, and miR-181a-3p, but it did downregulate the expression level of miR-181a-5p, which indicates the unique relationship between PBX1 and miR-181a-5p (Figure S4A-C). Taken together, we showed that PBX1, rather than ACAN, is a functional downstream target of miR-181a-5p that is responsible for the osteogenic effect of miR-181a-5p in OPLL cells.

### miR-181a-5p inhibits PBX1 to promote osteogenesis through histone demethylation

Since PBX1 has been reported to modulate the osteoblastogenesis process through epigenetic regulation of osteogenesis-related genes 14, we first compared the epigenetic state of several important ossification-related genes between OPLL and PLL cell samples. By using chromatin immunoprecipitation (ChIP) followed by real-time PCR analysis, and we found that the histone modification level of the PBX1 promoter did not significantly change between OPLL and PLL cells (Figure S5A). The expression levels of OSX and OCN, two known osteogenesis-related genes regulated by PBX1, were significantly different between OPLL and PLL cells (Figure S5B); additionally, the histone acetylation and methylation levels of their promoters were also different (Figure S5C-D). The results indicated that the downregulation of PBX1 in OPLL may be due to posttranscriptional regulation, and the upregulation of osteogenesis-related genes may be related to epigenetic regulation in OPLL cells.

**Figure 6:**
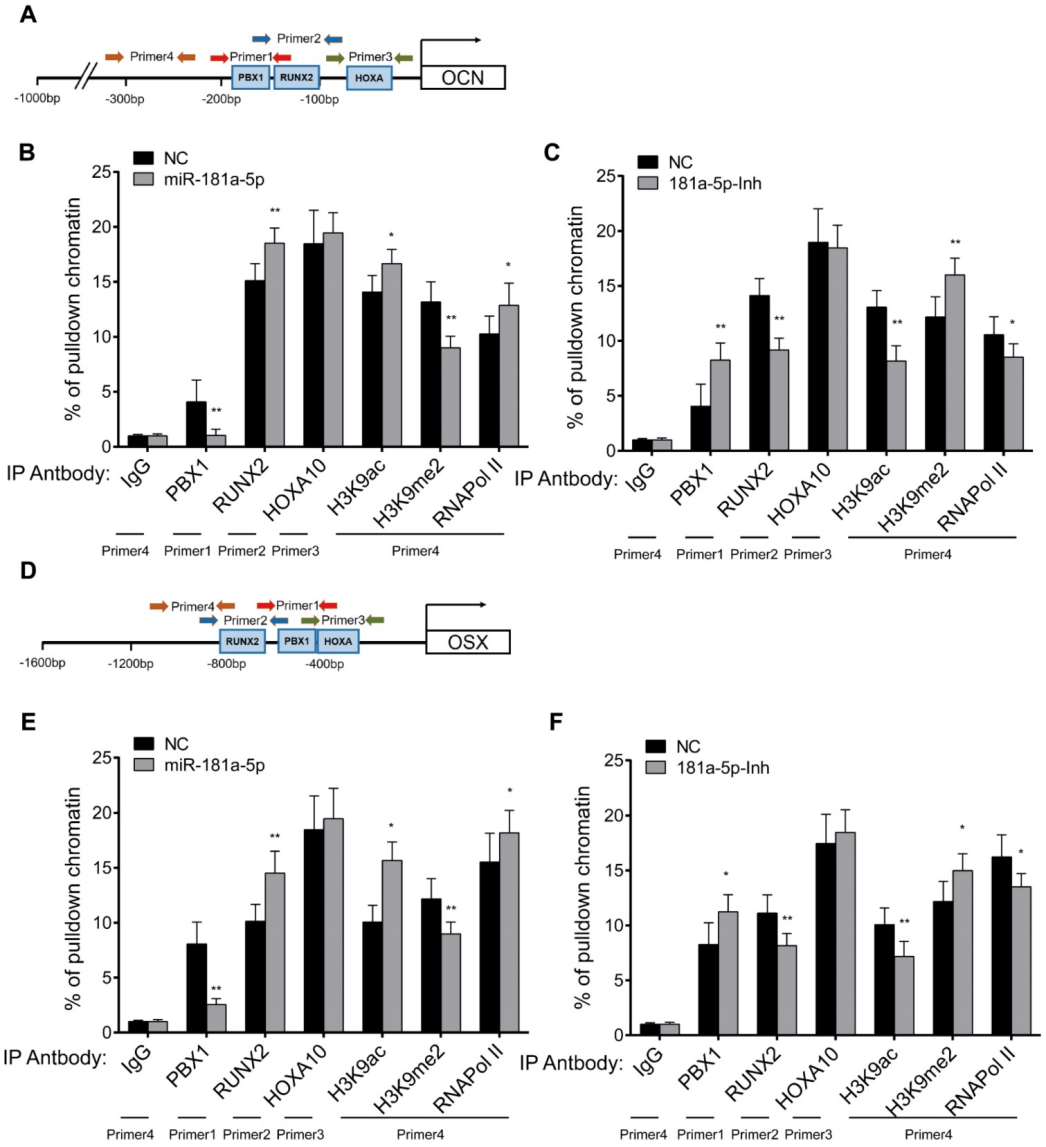
miR-181a-5p modulate the promoter histone modification level of Osx and Ocn gene through targeting PBX1. Jaspar predicted binding motif of PBX1, RUNX2 and HOXA10 to Ocn (A) gene promoter region. Primers used in ChIP assay are depicted in the graph. ChIP-PCR analysis showing the pulldown percentage of chromatin in Ocn promoter region by respective antibodies in miR-181-5p overexpressed OPLL cells (B) or miR-181-5p inhibited OPLL cells (C). Jaspar predicted binding motif of PBX1, RUNX2 and HOXA10 to Osx (D) gene promoter region. Primers used in ChIP assay are depicted in the graph, and ChIP-PCR analysis showing the pulldown percentage of chromatin in Osx promoter region by respective antibodies in miR-181-5p overexpressed OPLL cells (E) or miR-181-5p inhibited OPLL cells (F). IgG group was used as a negative control. All PCR experiments were repeated three times individually. All data were presented as the mean ± SD. *P < 0.05, **P < 0.01.

To further validate this hypothesis, we analyzed the histone modification level of the promoters of OCN and OSX using ChIP-PCR analysis (Figure 6A, D). Here, we also analyzed the binding of RUNX2 and HOXA10 to the promoters of OSX and OCN, as PBX1 is reported to repress the binding of RUNX2/HOXA10 by histone methylation 14, 15. In comparison to control levels, the epigenetic histone repression marker H3K9me2 was significantly reduced (Figure 6B, 6E), and the histone activation marker H3K9ac was significantly increased in the promoters of OCN and OSX (Figure 6B, 6E) after miR-181a-5p overexpression. Inverse changes were observed in the miR-181a-5p inhibition group (Figure 6C, 6F). The binding of RUNX2 to the OCN and OSX promoters was significantly increased after miR-181a-5p overexpression, but it was significantly reduced after miR-181a-5p inhibition (Figure 6B-C and Figure 6E-F). Taken together, we found that miR-181a-5p could modulate histone modification levels by regulating PBX1.

### miR-181a-5p promotes posterior longitudinal ligament ossification *in vivo*

**Figure 7:**
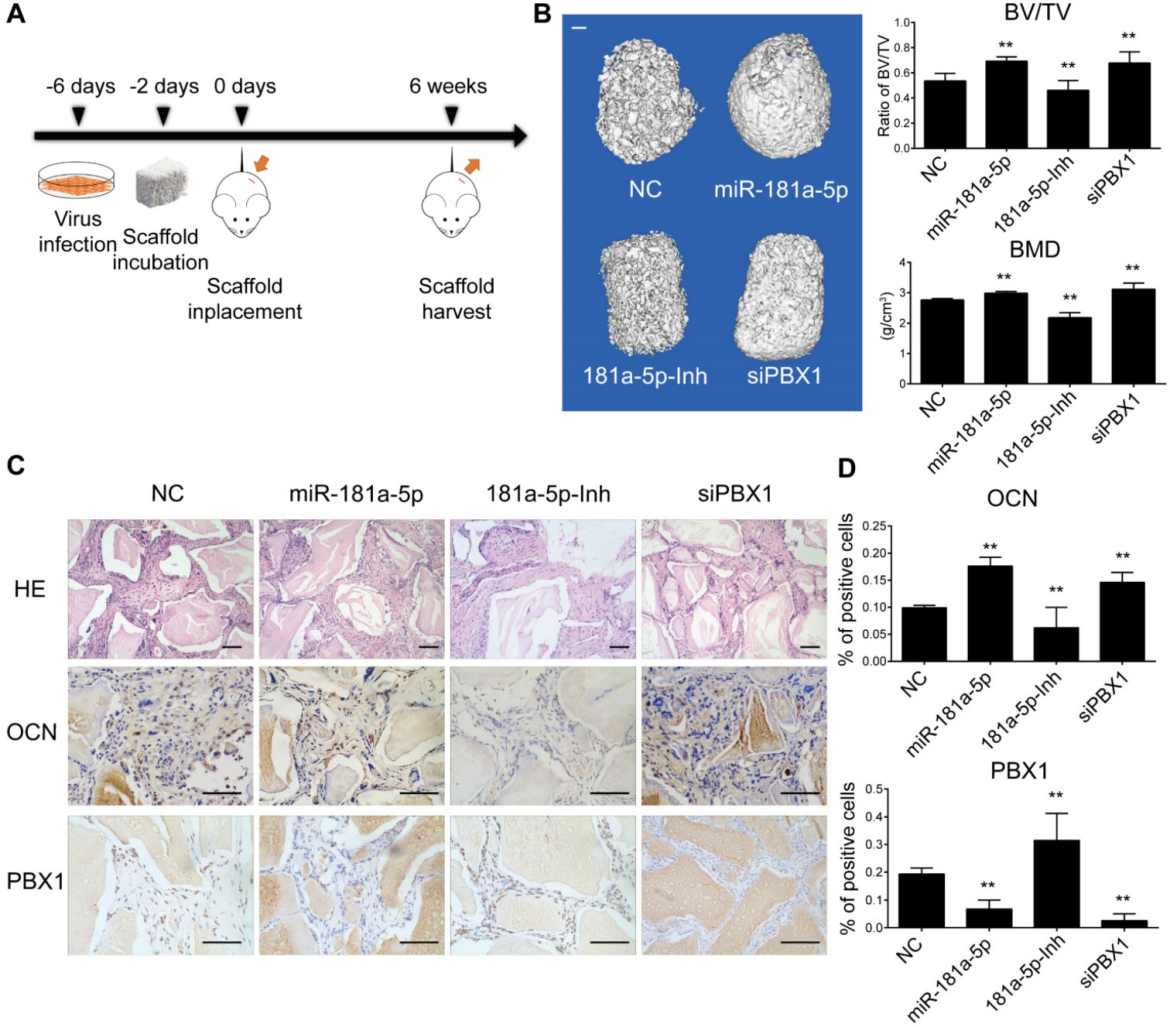
miR-181a-5p promoted heterotopic bone formation *in vivo*. (A) Scheme for heterotopic bone formation assay procedure. Lentivirus bearing miR-181a-5p overexpression or inhibition or PBX1 inhibition sequence were transfected to OPLL cells, and the cells were selected for stably expressing these sequences taking advantage of the puromycin antibiotic resistance expressed in the lentivirus. (B) Left panel: reconstructed three-dimensional micro-CT images of implanted bio-scaffold after 6 weeks. Right panel: percentages of new BV/TV and BMD of cultured bone constructs (n=6). The bar represents 500μm. (C) H&E staining and immunohistochemical staining of implanted bio-scaffold after 6 weeks in respective groups. The bar represents 150μm. (D) Quantification of OCN and PBX1 expression in the immunohistochemical staining of implanted bio-scaffold after 6 weeks (n=6). All data were presented as the mean ± SD. *P < 0.05, **P < 0.01.

To further analyze the function of miR-181a-5p *in vivo*, we first performed a heterotopic bone formation assay using nude mice. OPLL cells stably expressing miR-181a-5p, a miR-181a-5p inhibitor and a PBX1 knockdown siRNA were cocultured with Bio-Oss Collagen scaffolds for 2 days, and then the scaffolds were implanted subcutaneously on the back of nude mice (n=5) and allowed to grow for 6 weeks (Figure 7A). Micro-computed tomography (micro-CT) was used to detect the bone mass and the bone volume of the samples (Figure 7B). The ratio of bone volume/tissue volume (BV/TV) and bone mineral density (BMD) were significantly increased in the miR-181a-5p overexpression and PBX1 knockdown groups, and they were decreased in the miR-181a-5p inhibition group. Histological examination further confirmed the findings that the miR-181a-5p overexpression and PBX1 knockdown groups formed more lamellar bone tissue, and less lamellar bone tissue was formed in the miR-181a-5p inhibition group (Figure 7C). Immunohistochemistry staining of OCN and RUNX2 also showed similar results, in which miR-181a-5p overexpression and PBX1 knockdown resulted in more positively stained cells than were observed in the miR-181a-5p inhibition group (Figure 7C-D). These results indicated that miR-181a-5p can strongly promote ligament cell ossification *in vivo*.

**Figure 8:**
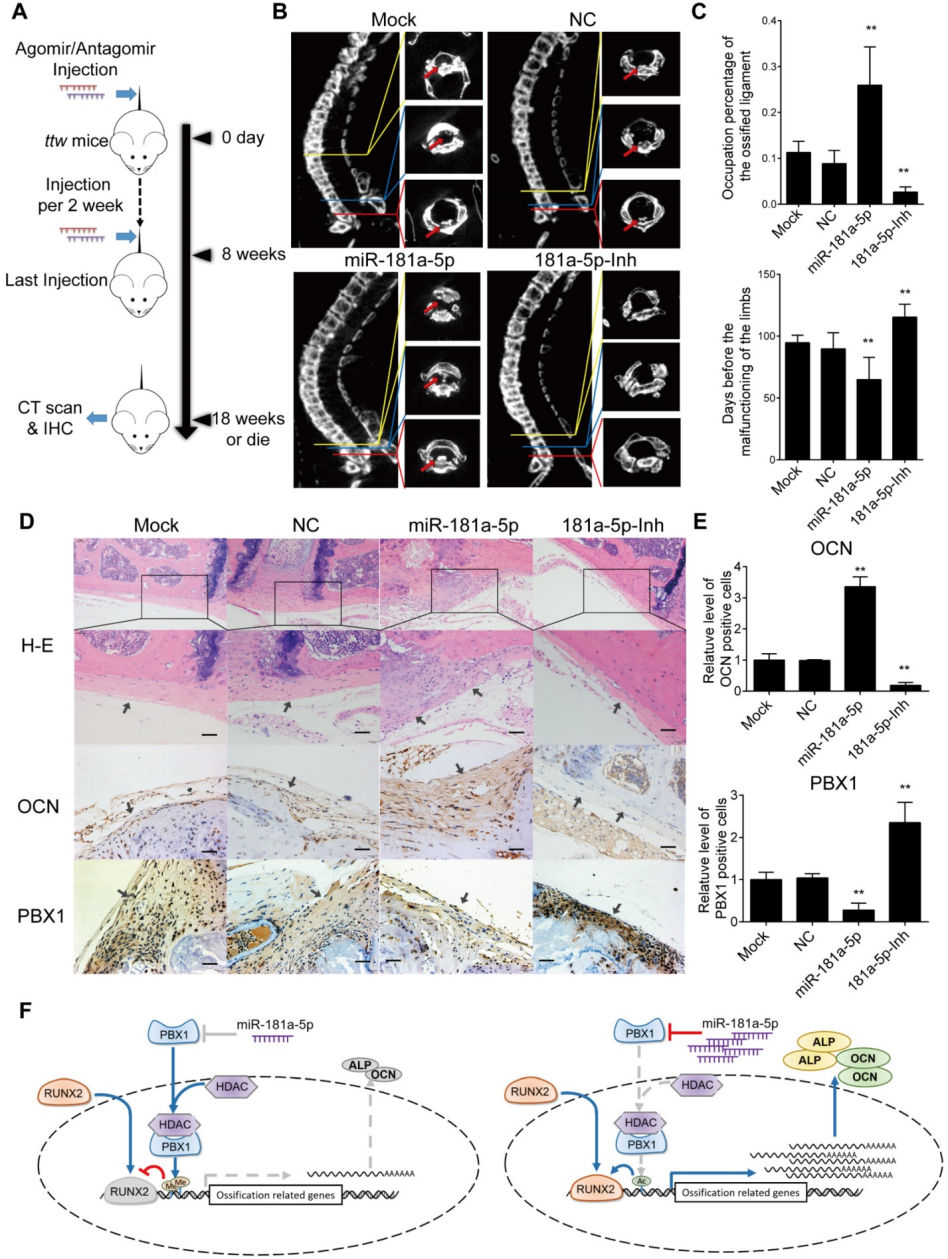
miR-181a-5p promoted OPLL development in *ttw* mice. (A) Experimental outline of miR-181a-5p agomir or antagomir tail vein injections to explore their effect in* ttw* mice. Injections were performed once every 2 weeks for 8 weeks. Mouse were sacrificed until 10 weeks after last injection and the spines were harvested, otherwise housed to observe the limbs functions. (B) micro-CT images of spine harvested spines from *ttw* mice in various groups. (C) The occupation percentage of the ossified mass in the spinal canal of *ttw* mice were compared (upper panel). And the symptom free interval is calculated and compared (lower panel). Any malfunctioning of the four limbs were determined as symptomatic. (D) H&E staining and immunohistochemical staining of the spine samples from *ttw* mice were analyzed, and the quantification of OCN and PBX1 expression in the immunohistochemical staining were shown (E). The bar represents 60μm. (F) The conclusive graph illustrating the molecular mechanism of miR-181a-5p in regulating OPLL development. All data were presented as the mean ± SD. *P < 0.05, **P < 0.01.

However, the effect of miR-181a-5p in OPLL disease has not been elucidated. Therefore, taking advantage of a known OPLL disease model, ttw (tip-toe-walking) mice, we performed systematic injections of miR-181a-5p or its inhibitor to investigate their effects (Figure 8A). Micro-CT was used to analyze the presence of ossified ligament mass and the percentage of the spinal canal that the mass has occupied (Figure 8B-C). We found that the miR-181a-5p injection group showed larger osteophytes in the posterior longitudinal ligament region and a higher spinal canal occupation rate than the other groups. In contrast, miR-181a-5p inhibition showed almost diminished osteophytes and the lowest spinal canal occupation rate of all groups. Similarly, the neurological symptom-free interval of the miR-181a-5p overexpression group was significantly reduced compared to that of the other groups (Figure 8C). Histochemistry analysis showed a thickened ligament accompanied by an upregulated OCN protein level and a reduced level of PBX1 in the miR-181a-5p overexpression group, while a relatively normal ligament and significantly increased PBX1 expression were observed in the miR-181a-5p inhibition group (Figure 8D-E). Consistent with our previous findings, we showed that increased miR-181a-5p expression within posterior longitudinal ligament cells could robustly repress PBX1 expression to increase histone deacetylase and decrease histone methylation at the promoter region of osteogenic genes, thus promoting the binding of RUNX2 to its downstream factors, which lead to ossification within the posterior longitudinal ligament (Figure 8F).

## Discussion

OPLL is a common spinal disease that can lead to symptoms of spinal cord compression and radiculopathy as the result of a mass of ectopic ossification developing in the posterior longitudinal ligament of the spine 16. Since no effective measures have been developed to inhibit or reverse the progression of the disease, it is urgent to find critical disease regulatory targets for developing gene therapies 17. In this study, by using *in vitro* experiments and *in vivo* OPLL models, we uncovered the function and mechanism of OPLL-related miR-181a-5p. We have shown that miR-181a-5p promotes ligament cell ossification both *in vitro* and *in vivo* and that systematic inhibition of miR-181a-5p using antagomirs manifests promising therapeutic potential in the OPLL mouse model. The data we presented suggest that miR-181a-5p inhibition may represent a functional treatment that needs further clinical validation.

Although both genetic and environmental (nongenetic) factors have been reported to be associated with the occurrence of OPLL, the specific mechanism is not clearly known. Various studies have shown that SNPs in osteogenic genes are associated with the onset of OPLL, and further characterization is required to link the SNPs with evident functional relationships associated with the occurrence or progression of the disease. The identified SNPs mostly reside in ossification-related genes, and these genes also contributed to normal skeletal development or related diseases. Thus, we speculate that these factors are important “effectors” rather than “activators” of OPLL development. In previous studies, we revealed that miRNAs are vital factors that link many osteogenic genes with OPLL 9, 18. Due to the pathophysiology of OPLL, we hypothesized that miRNAs may be important upstream factors that control the osteogenic process by regulating ossification-related genes in OPLL. In this study, we provide evidence to support this hypothesis by constructing an ossification-related miRNA/mRNA interactive network constructed with ossification-related genes that were differentially expressed in OPLL 9, 19. The ossification-related miRNA/mRNA interactive network showed a more intense regulatory relationship between the genes, among which miR-218-5p, miR-196a-5p, miR-330-3p and miR-181a-5p had more clustered ossification-related targets, which implies a potential role for these microRNAs (and their targets) in OPLL development. Many microRNAs in the network have been previously reported to be associated with osteogenesis, such as miR-218-5p and miR-196a-5p 20-22. During the study, we selected these ossification-related microRNAs as candidates and for functional testing during ligament cell osteogenesis, and of those that we tested, miR-218-5p, miR-196a-5p, miR-330-3p and miR-181a-5p, only miR-181a-5p showed significant upregulation of the expression of ossification-related RUNX2, ALP and OSX genes (data not shown); this result is similar to that of miR-10a-3p, which we verified previously 12. This phenomenon implies that the ossification process in the posterior longitudinal ligament may regulate genes differently than other tissues, as some of the reported ossification-related microRNAs did not play a significant role in the process.

The miR-181a that we studied is in a conserved microRNA family that contains six isoforms: mir181a-1/2, mir181b-1/2 and mir181a-c/d. They are involved in many biological processes, such as signal transduction, ontogeny and carcinogenesis 23. Reports have shown that miRNA-181 is essential for the onset of embryo implantation by directly targeting leukemia inhibitory factor (LIF) and downregulating its expression during embryo implantation 23. In tumor development, miR-181 was reported to act as a tumor suppressor in the pathogenesis of acute myeloid leukemia (AML), where it exhibits a significant impact on the survival of patients with AML 24. Others have found that miRNA-181 is significantly upregulated in differentiated skeletal muscle and that it can promote differentiation 25. In skeletal development, studies have shown that miRNA-181a acts as an osteogenic promotor via repression of TGF-β signaling in MC3T3 cells 26. Our data confirmed the upregulation of miR-181a during ligament cell ossification, while miR-181a-5p, but not miR-181a-3p, functioned as the main modulator during osteogenesis in human posterior longitudinal ligament cells. Although involved in many biological processes, the mechanism differs greatly between cell lines and pathological conditions. Our findings showed that miR-181a-5p could modulate the epigenetic histone modification state of the ligament cells through regulation of PBX1, a reported histone modification regulator in osteogenesis. This function of miR-181a is new, and this is the first article to decipher the epigenetic regulatory network of OPLL to date. Our findings here further expanded the functions of miR-181.

The pre-B-cell leukemia homeobox protein (Pbx) family includes Pbx1, Pbx2, Pbx3 and Pbx4, which are evolutionarily conserved, atypical homeodomain proteins. They belong to the three-amino-acid loop extension (TALE) family of homeodomain-containing transcription factors. Many studies have found them to be of great importance to multiple cell fate decisions. Pbx1, but not Pbx2 and 3, plays a critical role in skeletal development and chondrocyte proliferation and differentiation 27. Transient gene silencing of Pbx1 in murine MC3T3-E1 preosteoblasts enhanced cell proliferation and bone nodule formation 28. Pbx1 overexpression increased ossification and markers of mature osteoblasts, such as Ocn and Bsp, in C3H10T1/2 cells 14. These results indicate that Pbx1 is a negative regulator of ossification. Pbx binds to DNA with other cofactors to regular the expression of genes, such as HOX, MEIS, MyoD, and FOX 15, 29, 30. In addition, Pbx is involved in regulating transcription through histone modification, including acetylation and methylation 31. In the present study, by means of a functional assay, we found that miR-181a-5p exerts its ossification function by downregulating the expression of PBX1, and we found that PBX1 is also an important negative regulator of ligament cell ossification. The molecular mechanism is partly due to PBX1-mediated H3K9me2 and H3K9ac modifications in the promoter regions of ossification-related genes such as OSX and OCN. However, we did not find direct binding of PBX1 to the promoter region of RUNX2 and ALP in the ChIP assay, which implies that the expression changes of these two factors during the ossification process of OPLL may be due to the consequent regulation of other ossification master genes, such as OSX.

To verify the *in vivo* function of miR-181a-5p, we used two approaches: heterotopic bone formation assays and analysis of an OPLL disease model. For the OPLL disease model, we used tip-toe-walking mice (TWY-ttw mice) (CIEA, Kawasaki, Japan). This model is known for its development of soft tissue ossification after birth, and studies have claimed it to be a suitable model for OPLL study 32, 33. We observed that ttw mice as early as 12 weeks old developed limb paralysis, meaning they could not drink water under normal circumstances. By injecting miR-181a-5p-overexpressing agomirs or antagomirs, we found that the osteophyte formed in the spinal canal is greatly affected, and we further confirmed that the miR-181a-5p/PBX1 regulatory axis is functional in OPLL development, as shown by IHC analysis of the spinal tissues from treated ttw mice. Although the functional role of the miR-181a-5p/PBX1 regulatory axis in OPLL was revealed in this study, the upstream factors of the axis are still unknown. PBX1 is a well-known pioneer transcription factor that controls the transcription of cell fate genes 31, but less is known about regulators of its own expression. Here, we showed that miR-181a-5p could regulate PBX1 expression posttranscriptionally. Together, these data introduce the possibility that miR-181a-5p inhibitor antagomirs could be further exploited for the development of therapeutic agents against OPLL.

## Conclusion

Our study unveiled the mechanism by which microRNA-181a regulates the ossification process of OPLL ligament cells by modulating the histone modification level of ossification-related genes through direct targeting of PBX1. Our data also showed the therapeutic effects of miR-181a antagomir in preventing OPLL development both *in vivo* and *in vitro*. Our work is the first to demonstrate that microRNA perturbation could modulate the development of OPLL through epigenetic regulation, which may shed light on the development of therapeutic agents against OPLL.

## Abbreviations

PLL: posterior longitudinal ligament
OPLL: ossification of posterior longitudinal ligament
PCR: polymerase chain reaction
RUNX2: RUNX family transcription factor 2
OCN: osteocalcin
OSX: osterix
ChIP: Chromatin immunoprecipitation
PBX1: pre-B-cell leukemia homeobox protein
GAPDH: glyceraldehyde 3-phosphate dehydrogenase
ALP: alkaline phosphatase
HO: heterotopic ossification
*ttw*: tiptoe walking mouse
UTR: untranslated region
