# Supplementary material for: MicroRNA-181 regulates the development of Ossification of Posterior longitudinal ligament via Epigenetic Modulation by targeting PBX1
Source: Theranostics. 2020 Jun 12;10(17):7492–509. doi: 10.7150/thno.44309 (PMC7359103; doi:10.7150/thno.44309)
Supplement: Supplementary file 1 — Supplementary figures and tables. [file thnov10p7492s1.pdf]

Supplementary Materials

MicroRNA-181 Regulate the Development of Ossification of Posterior Longitudinal Ligament via Epigenetic Modulation by Targeting PBX1

Authors: Ning Liu[1, *], Zicheng Zhang[2, *], Xiaolong Shen[1, *], Li Li[3, *], Baifeng Sun[1], Ruizhe Wang[1], Huajian Zhong[1], Qianghui Shi[1], Leixin Wei[1], Yizhi Zhang[1], Yue Wang[3], Chen Xu[1, #], Yang Liu[1, #], Wen Yuan[1, #]

Affiliations: [1] Department of Orthopedics, Changzheng Hospital Affiliated to Second Military Medical University, 415th Feng Yang Road, Shanghai, 200003, PR China.

[2]Undergraduate Brigade, Changhai Hospital Affiliated to Second Military Medical University, 168th Chang Hai Road, Shanghai, 200433, China

[3] Research Center of Developmental Biology, Second Military Medical University, 800th Xiang Yin Road, Shanghai, 200433, PR China.

[#] Corresponding authors

* These authors contributed equally to this work

Contact Information of Corresponding Authors:

Prof. Chen Xu, Tel and Fax: +86-021-81870958, E-mail: chenxu8836@hotmail.com

Prof. Yang Liu, Tel and Fax: +86-021-81870958, E-mail: yangliuchz@hotmail.com

Prof. Wen Yuan, Tel and Fax: +86-021-81870965, E-mail: yuanwenspine@smmu.edu.cn

Supplementary Tables

Table S1 Demographic data of the patients involved in the study

	PLL	OPLL	P value
**Number of cases**	19	22	>0.05
**Mean Age (years)**	55.2±8.2	57.7±9.2	>0.05
**Gender (Male:Female)**	11:8	13:9	>0.05
**BMI**	25.7±6.2	26.3±7.2	>0.05
**Type of OPLL**			
local	-	-	-
segmental	-	3	-
continuous	-	14	-
mixed	-	5	-
**Other diseases**			
Diabetes	1	2	>0.05
Hypertension	2	4	>0.05
DISH	-	-	-
Fibrodysplasia ossificans progressive	-	-	
Myositis ossificans	-	-	
OLF	-	-	-
CAD	-	-	-

Data were presented as Mean±S.D., OLF, Ossification of the ligamentum flavum; DISH, diffusive idiopathic hyperostosis; CAD, Coronary artery disease

Supplementary Videos

Supplementary Video 1. Typical ttw mice with OPLL symptoms and ossified ligament tissue present in the spine canal.

Supplementary Data

Supplementary Data 1. The Ossification related miRNA/mRNA negative correlation network data in OPLL (GSE69787)

Supplementary Data 2 Oligonucleotide Sequences used in this study

Supplementary Figures & Legends

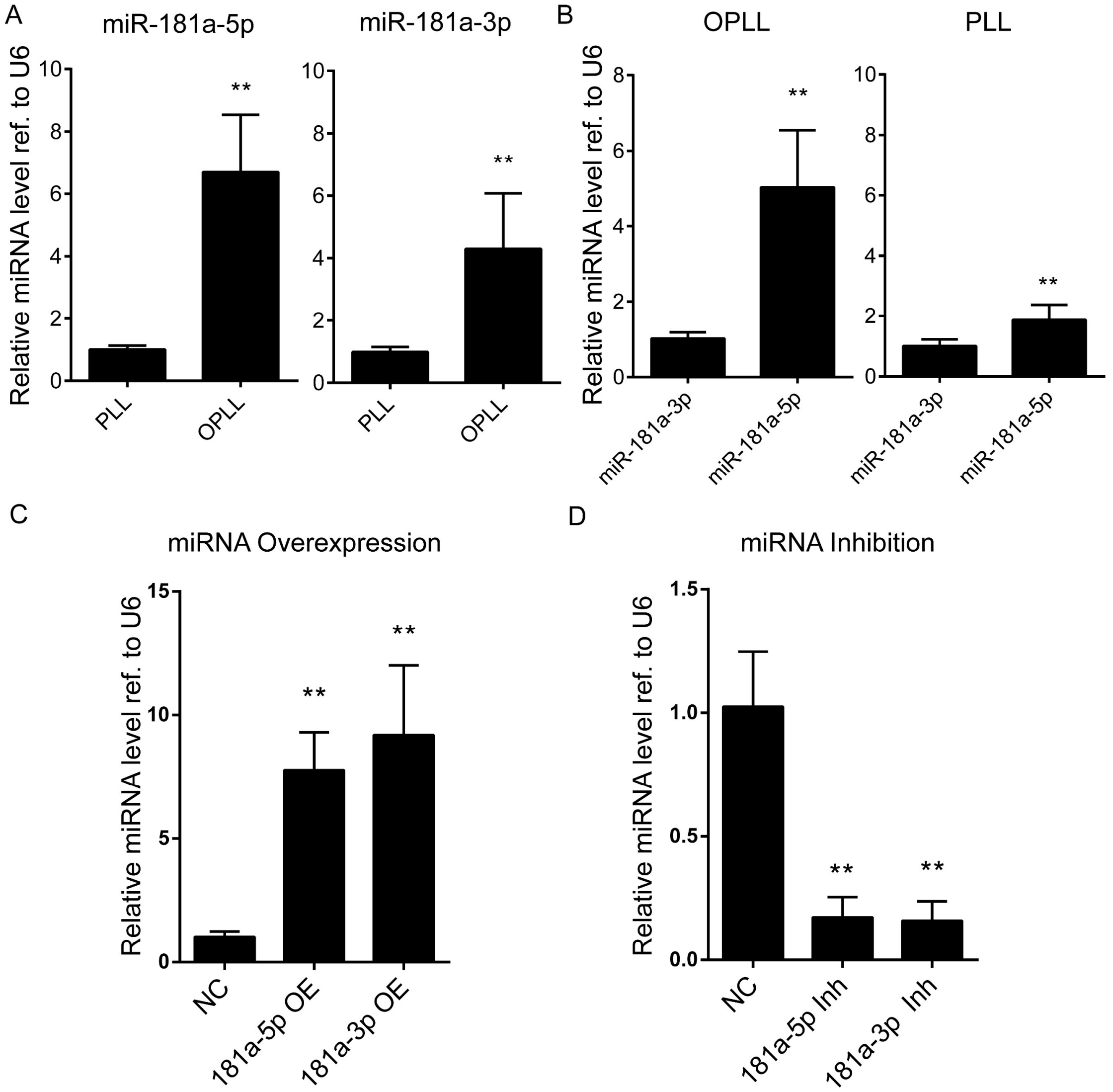

Supplementary Figure S1. (A) The expression level of miR-181a-5p and -3p were analyzed and compared between PLL and OPLL cells using real-time PCR analysis. (B) Real-time PCR analysis comparing the expression abundance between miR-181a-5p and -3p in OPLL or PLL cells. We can see that the expression level of miR-181a-5p is significantly higher than miR-181a-3p in OPLL cells. (C) MicroRNA overexpression efficiency is tested using real-time PCR analysis in PLL cells. (D) MicroRNA inhibition efficiency is tested using real-time PCR analysis in OPLL cells. All PCR experiments were repeated three times individually, and U6 level were detected and served as internal reference. All data were presented as the mean ± SD. *P < 0.05, **P < 0.01.

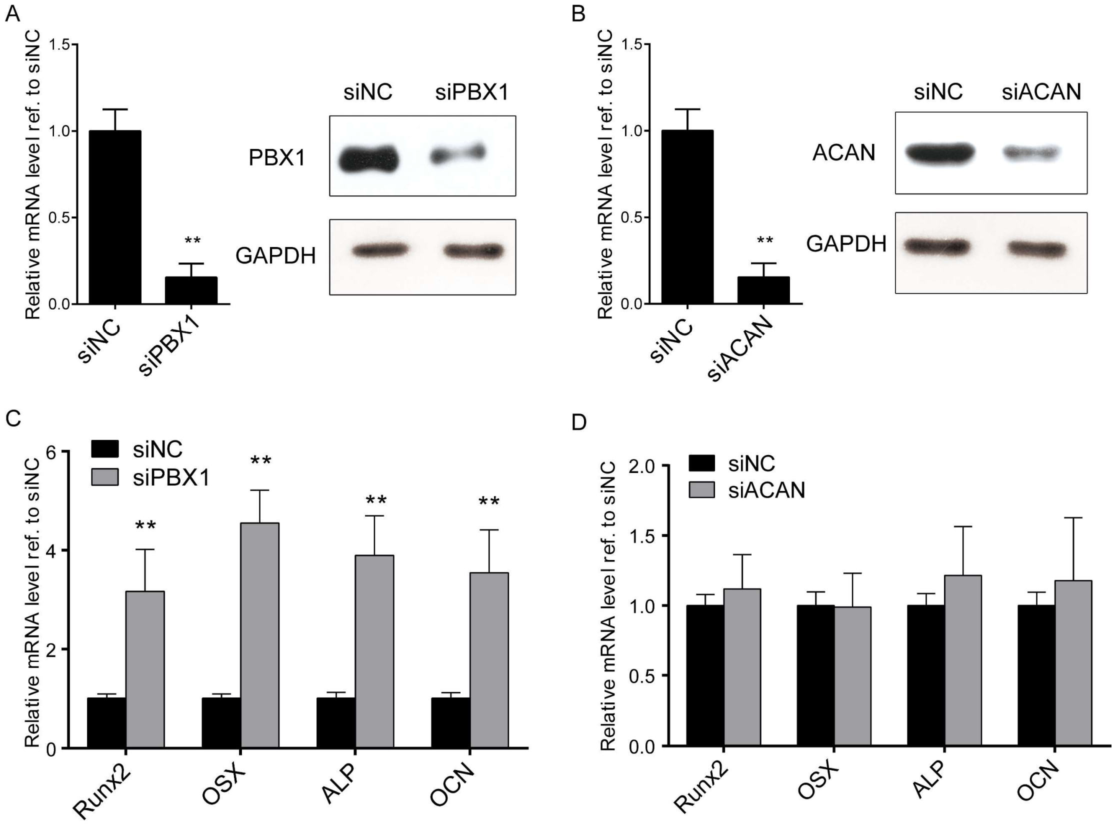

Supplementary Figure S2. (A) Knockdown efficiency of PBX1 targeting siRNA was analyzed in PLL cells using real-time PCR analysis and Western blot. (B) Knockdown efficiency of ACAN targeting siRNA was analyzed in PLL cells using real-time PCR analysis and Western blot. Real-time PCR analysis showing the mRNA expression level of osteogenic genes after knockdown of PBX1 (C) or ACAN (D) using small interference RNAs in PLL cells. The siNC represents transfecting scramble control siRNAs which serve as control group. All PCR experiments were repeated three times individually, and GAPDH level were detected and served as internal reference. All data were presented as the mean ± SD. *$P < 0.05$, **$P < 0.01$.

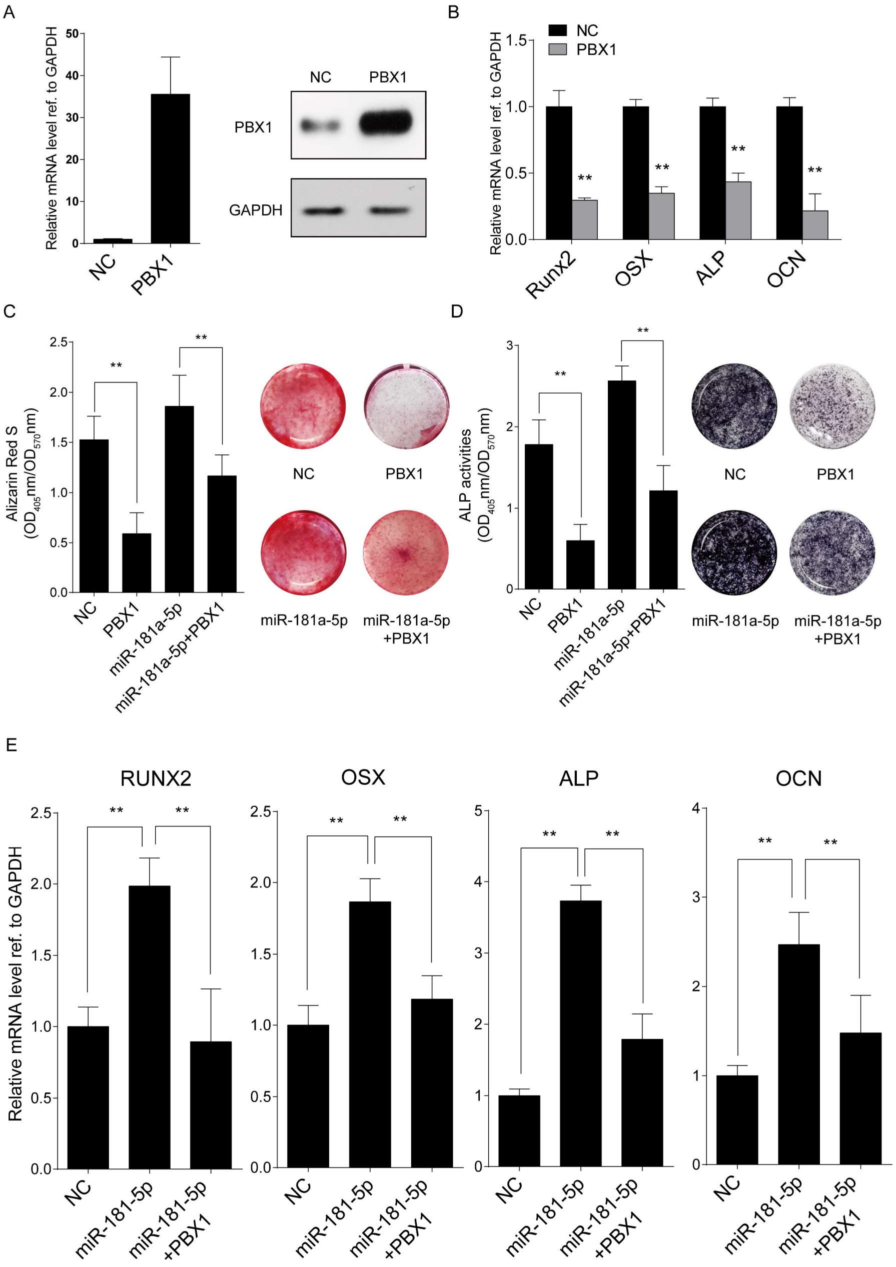

Supplementary Figure S3. (A) Overexpression efficiency of PBX1 overexpressing lentivirus was analyzed in OPLL cells using real-time PCR analysis and Western blot. (B) Real-time PCR analysis showing the mRNA expression level of osteogenic genes after overexpression of PBX1 in OPLL cells during osteogenic induction. Alizarin red staining (C) or alkaline phosphatase staining (D) analyses to show the osteogenic properties of respective treatment in OPLL cells during osteogenic induction. The colorimetric quantification is shown in the right panels, respectively. (E) Real-time PCR analysis was used to analysis mRNA expression levels of osteogenic genes in various groups in OPLL cells. All PCR experiments were repeated three times individually, and GAPDH level were detected and served as internal reference. All data were presented as the mean ± SD. *P < 0.05, **P < 0.01.

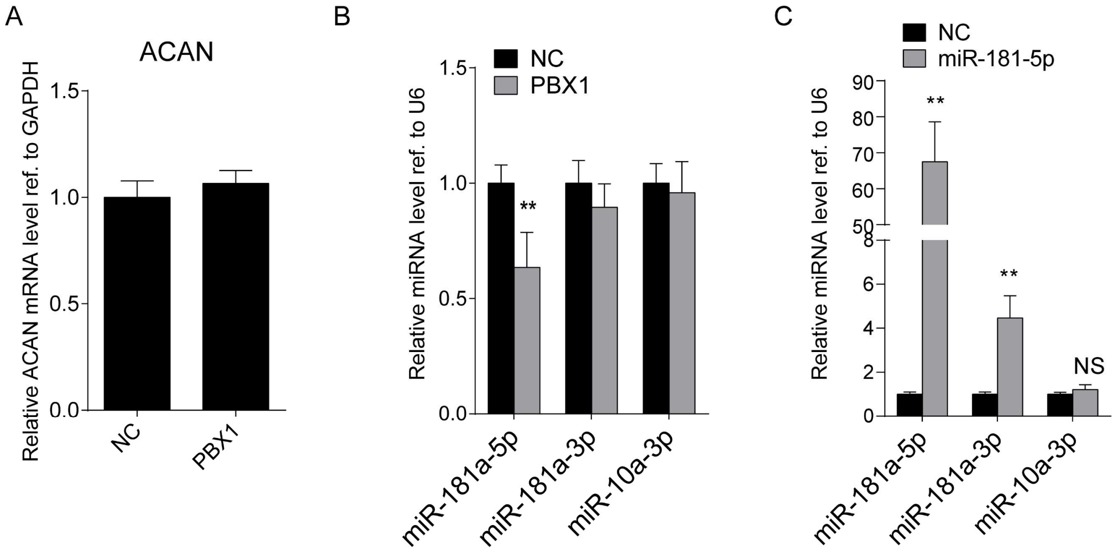

Supplementary Figure S4. (A) Real-time PCR analysis detecting ACAN expression after PBX1 overexpression in OPLL cells during osteogenic induction. (B) Real-time PCR analysis detecting miR-181a-5p, -181a-3p, -10a-3p expression after PBX1 overexpression in OPLL cells during osteogenic induction. (C) Real-time PCR analysis detecting miR-181a-5p, -181a-3p, -10a-3p expression after miR-181a-5p overexpression in OPLL cells during osteogenic induction. All PCR experiments were repeated three times individually, GAPDH and U6 level were detected and served as internal reference (GAPDH for mRNA, U6 for miRNA). All data were presented as the mean ± SD. *P < 0.05, **P < 0.01.

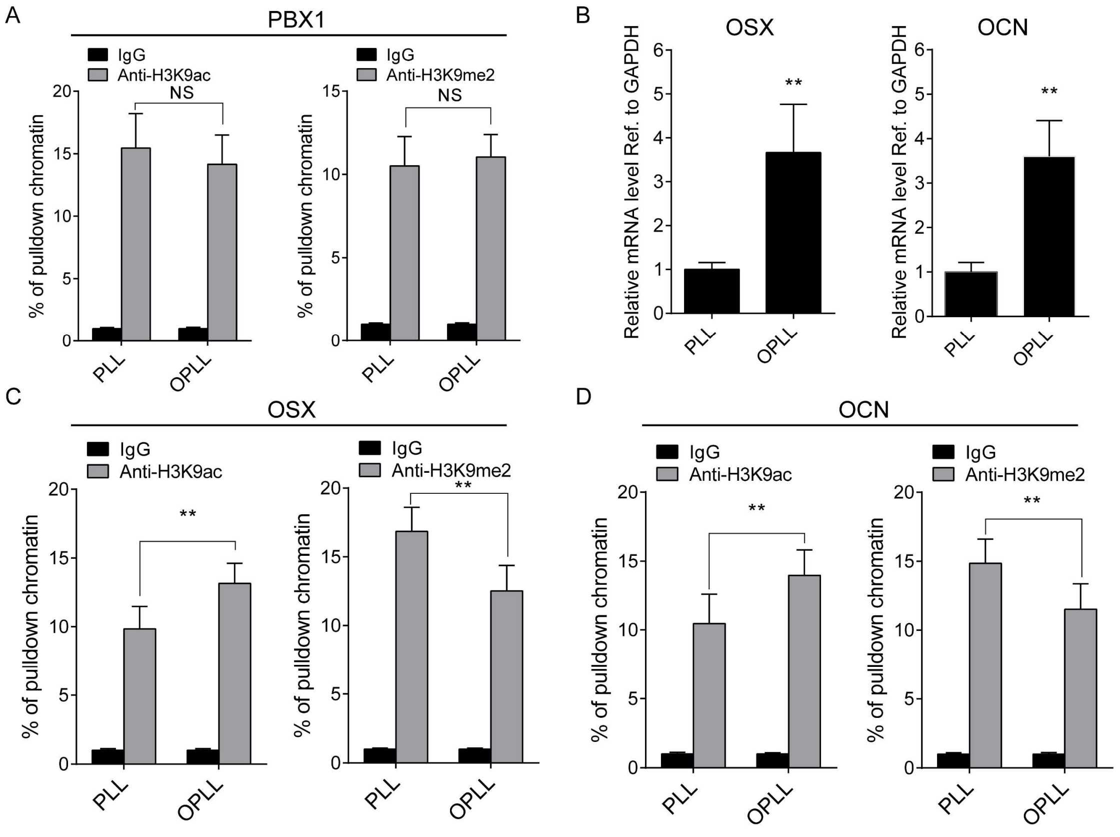

Supplementary Figure S5. (A) ChIP-PCR analysis showing the pulldown percentage of chromatin in PBX1 promoter region by respective histone marker antibodies in PLL or OPLL cells. (B) The expression level of OSX and OCN in PLL or OPLL cells were analyzed using real-time PCR analysis. ChIP-PCR analysis showing the pulldown percentage of chromatin in OSX (C) or OCN (D) promoter region by respective histone marker antibodies in PLL or OPLL cells. All PCR experiments were repeated three times individually, and GAPDH level were detected and served as internal reference. All data were presented as the mean ± SD. *P < 0.05, **P < 0.01.
